# An Estimation of the Levels of Stabilized Criegee Intermediates in the UK Urban and Rural Atmosphere Using the Steady‐State Approximation and the Potential Effects of These Intermediates on Tropospheric Oxidation Cycles

**DOI:** 10.1002/kin.21101

**Published:** 2017-06-12

**Authors:** M. Anwar H. Khan, William C. Morris, Matthew Galloway, Beth M. A. Shallcross, Carl J. Percival, Dudley E. Shallcross

**Affiliations:** ^1^ Atmospheric Chemistry Research Group School of Chemistry University of Bristol Bristol BS8 1TS UK; ^2^ School of Pharmacy The University of Manchester Manchester, M13 9PL UK; ^3^ NASA Jet Propulsion Laboratory Pasadena CA 91109

## Abstract

Levels of the stabilized Criegee Intermediate (sCI), produced via the ozonolysis of unsaturated volatile organic compounds (VOCs), were estimated at two London urban sites (Marylebone Road and Eltham) and one rural site (Harwell) in the UK over the period of 1998–2012. The steady‐state approximation was applied to data obtained from the NETCEN (National Environmental Technology Centre) database, and the levels of annual average sCI were estimated to be in the range of 30–3000 molecules cm^−3^ for UK sites. A consistent diurnal cycle of sCI concentration is estimated for the UK sites with increasing levels during daylight hours, peaking just after midday. The seasonal pattern of sCI shows higher levels in spring with peaks around May due to the higher levels of O_3_. The ozone weekend effect resulted in higher sCI in UK urban areas during weekend. The sCI data were modeled using the information provided by the Air Quality Improvement Research Program (AQIRP) and found that the modeled production was five‐ to six‐fold higher than our estimated data, and therefore the estimated sCI concentrations in this study are thought to be lower estimates only. Compared with nighttime, 1.3‐ to 1.8‐fold higher sCI exists under daytime conditions. Using the levels of sCI estimated at Marylebone Road, globally the oxidation rates of NO_2_ + sCI (22.4 Gg/yr) and SO_2_ + sCI (37.6 Gg/yr) in urban areas can increase their effect in the troposphere and potentially further alter the oxidizing capacity of the troposphere. Further investigations of modeled sCI show that CH_3_CHOO (64%) and CH_2_OO (13%) are dominant among all contributing sCI at the UK sites.

## INTRODUCTION

Criegee Intermediates (CI) can be formed from the ozonolysis of alkenes with a range of energies and those termed “stabilized” Criegee intermediates (sCI) have less energy than is required to decompose rapidly and may have lifetimes of sufficient length that they can react with coreactants in the atmosphere 1.

(1) Alkene +O3→α CI +(1-α) sCI + carbonyl

where CI can undergo rapid unimolecular decomposition in the main with a branching fraction of α, and the remaining is the stabilized CI with a branching fraction of (1–α). The decomposition of the CI gives a number of products including OH, CO_2_, and hydrocarbons. The sCI can react with various atmospheric compounds, “Criegee scavengers,” including H_2_O, water dimer ((H_2_O)_2_), NO_2_, SO_2_, alcohols, aldehydes, and carboxylic acids, thus these sCI can play a significant role in controlling the budget of many tropospheric species (e.g., HO*_x_*, NO*_x_* (NO + NO_2_), NO_3_, O_3_, SO_2_, H_2_SO_4_, organic acids, carbonyl compounds, secondary organic aerosol) 2, 3, 4, 5, 6, 7, 8, 9, 10, 11, 12, 13, 14, 15, 16, 17, 18, 19.

There is an uncertainty about the loss processes of sCI, with the highly variable estimated unimolecular thermal loss within a range between 3 and 360 s^−1^ (Table I). In the atmosphere, it is likely that H_2_O, (H_2_O)_2_, SO_2_, NO_2_, and carboxylic acids are of appreciable concentrations to act as the sinks of sCI and indeed H_2_O for most sCI and (H_2_O)_2_ for CH_2_OO are believed to be the dominant sinks. The rate constants from the reactions of sCI + H_2_O and sCI + (H_2_O)_2_ range from 1.0 × 10^−14^ to 2.0 × 10^−19^ cm^3^ molecule^−1^ s^−1^ and 1.6 × 10^−11^ to 2.6 × 10^−14^ cm^3^ molecule^−1^ s^−1^, respectively (Table I). The rate coefficients range between (2.0–7.0) × 10^−12^ cm^3^ molecule^−1^ s^−1^ for sCI + NO_2_ reaction 7, 9, 22, 26, (2.4–22.0) × 10^−11^ cm^3^ molecule^−1^ s^−1^ for sCI + SO_2_ reaction 7, 9, 15, 20, 22, 26, 29, 32, 33, and (1.0–6.0) × 10^−10^ cm^3^ molecule^−1^ s^−1^ for sCI + carboxylic acid reaction 19.

(2) $$\begin{array}{c} \mathrm{sCI}\to \mathrm{Products} \end{array}$$

(3) $$\begin{array}{c} \mathrm{sCI}+H_{2}O\to \mathrm{Products} \end{array}$$

(4) $$\begin{array}{c} \mathrm{sCI}+{\left(H_{2}O\right)}_{2}\to \mathrm{Products} \end{array}$$

(5) sCI + NO 2→ NO 3+ carbonyl

(6) sCI + SO 2→ SO 3+ carbonyl

(7) $$\begin{array}{c} \mathrm{sCI}+\mathrm{RCOOH}\to \mathrm{Products} \end{array}$$

**Table I:** Rate Coefficients for the Unimolecular Loss of sCI and Their Reactions with Water and Water Dimer

Unimolecular Loss					
sCI	Rate Coefficient (s^−1^)	Reference			
CH_2_OO	11.6 ± 8.0	20			
	115 ± 20	23			
CH_3_CHOO	288 ± 275 (*syn*)	21			
	3–30 (*syn*)	17			
	≤250	9			
	76	24			
(CH_3_)_2_COO	305 ± 70	22			
	361 ± 49	25			

Loss by H_2_O			Loss by (H_2_O)_2_		
sCI	Rate Coefficient (cm^3^ molecule^−1^ s^−1^)	Reference	sCI	Rate Coefficient (cm^3^ molecule^−1^ s^−1^)	Reference
CH_2_OO	2.4 × 10^−16^	16	CH_2_OO	(6.5 ± 0.8) × 10^−12^	27
	3.7 × 10^−16^	28		5.4 × 10^−12^	28
	<4 × 10^−15^	7		(7.4 ± 0.6) × 10^−12^	30
	(2.5 ± 1.0) × 10^−17^	18		(4.0 ± 1.2) × 10^−12^	31
	<9 × 10^−17^	26			
	(1.3 ± 0.4) × 10^−15^	21			
	<1.5 × 10^−15^	27			
*syn*‐CH_3_CHOO	1.9 × 10^−19^	16	*syn*‐CH_3_CHOO	2.6 × 10^−14^	28
	2.0 × 10^−19^	28			
	<4 × 10^−15^	9			
	<2 × 10^−16^	29			
*anti*‐CH_3_CHOO	5.2 × 10^−15^	16	*anti*‐CH_3_CHOO	1.6 × 10^−11^	28
	3.4 × 10^−14^	28			
	(2.3 ± 2.1) × 10^−14^	21			
	(1.0 ± 0.4) × 10^−14^	9			
	(2.4 ± 0.4) × 10^−14^	29			
(CH_3_)_2_COO	(2.1 ± 0.6) × 10^−15^	21	(CH_3_)_2_COO	<1.3 × 10^−13^	15
	<1.5 × 10^−16^	15			

sCI is believed to provide an important source of NO_3_ through the reactions (1) and (5) 9, 18, 34, 35, which can be responsible for the formation of organic nitrates; these nitrates can be condensed in the urban areas, which could have a large impact on aerosol formation 36, 37, 38. Reaction (6) is an important reaction in the atmospheric cycle as SO_3_ can go on to produce H_2_SO_4_, which is an effective compound in the formation of aerosol particles 11, 12, 13.

The recent experimental evidence confirms the existence of sCI 7, 9, 15, 20, 22, 26, 28, 29, 31, 33, 39, 40, 41, but relatively little is known about its quantitative effect on the atmospheric cycle. The levels of anthropogenic volatile organic compounds (VOCs) (e.g., alkanes, alkenes, and aromatics) are expected to be elevated in the urban atmosphere because of their emissions from traffic and other combustion sources; these VOCs can then take part in photochemical ozone production in the downwind urban plume 42. The elevated concentrations of ozone along with increased levels of VOCs (alkenes) can lead to the formation of sCI in the urban environment. However, sCI are highly reactive and unstable making them difficult to isolate and measure in the atmosphere. Therefore, we have estimated sCI concentrations at two urban sites (e.g., Marylebone Road, Eltham), and one rural site (e.g., Harwell) in the UK over the time period of 1998–2012 using the steady‐state approximation method. Data for O_3_ and alkenes are taken from hourly measurements made as part of the NETCEN (National Environmental Technology Centre) data archive 43. The alkene data sets were modeled using information provided by the Air Quality Improvement Research Program (AQIRP) 44, resulting in an increase in the alkene data set from 9 to 52, and these extended numbers of alkenes were used to calculate model sCI concentrations in the UK urban and rural areas. The potential impacts of the level of sCI on the atmospheric cycle have also been discussed in the study.

## METHODOLOGY

### The Steady‐State Approximation

Assuming the steady‐state approximation of sCI, the rate of total production of sCI and the rate of loss of sCI are equal, i.e.,

$$\begin{array}{c} d[\mathrm{sCI}]/dt=0 \end{array}$$

Considering all the productions and losses of sCI, it is possible to construct a steady‐state equation to calculate the concentration of sCI

(I) [ sCI ]=∑k1[O3][ alkene ](1-α)k2+k3[H2O]+k4[(H2O)2]+k5[ NO 2]+k6[ SO 2]+k7[ RCOOH ]

Comparing the unimolecular thermal loss and the losses by H_2_O and (H_2_O)_2_, the loss processes via NO_2_, SO_2_, and RCOOH are negligible, so the loss process of sCI can be represented by *k_2_* + *k_3_*[H_2_O] + *k_4_*[(H_2_O)_2_]. Therefore, [sCI] now becomes

(II) $$\begin{array}{c} \left[\mathrm{sCI}\right]=\frac{\sum k_{1}\left[O_{3}\right]\left[\mathrm{alkene}\right]\left(\text{1-}\alpha \right)}{k_{2}+k_{3}\left[H_{2}O\right]+k_{4}\left[{\left(H_{2}O\right)}_{2}\right]} \end{array}$$

The available nine unsaturated hydrocarbons (1,3‐butadiene, 1‐butene, 1‐pentene, ethene, isoprene, propene, *cis*‐2‐butene, *trans*‐2‐butene, *trans*‐2‐pentene) and O_3_ hourly data were obtained via the NETCEN data base. The instrument used to monitor hydrocarbons was a Chrompack CP9000 VOCAIR system, a GC fitted with an automated thermos‐desorption/cryogenic trapping system. The standards supplied by the National Physical Laboratory (NPL) contained 26 component mixtures in nitrogen. The detailed description of the network, the VOCAIR system and the data handling, and analysis can be found in Dollard and co‐workers 45. There are some missing data in NETCEN database during the time period of 1998–2012 because of the instrument malfunctioning, instrumental maintenance, and routine calibration.

The rate coefficients used in the production of sCI from the reactions of alkenes with O_3_ can be found in the Supporting Information (Table S1). Using the fraction of sCI (1‐α) of 0.5 46, the production of all [sCI] was calculated. sCI was then split into CI‐1 (CH_2_OO), CI‐2 (RCHOO), and CI‐3 (R_2_COO). Taking account of the unimolecular loss rate (*k_2_*) of 11.6 s^−1^ for CI‐1 20, 250 s^−1^ for CI‐2 9, and 305 s^−1^ for CI‐3 22, the rate coefficient of the reaction with H_2_O (*k*_3_) of 2.4 × 10^−16^ cm^3^ molecules^−1^ s^−1^ for CI‐1 16, 1.9 × 10^−19^ cm^3^ molecules^−1^ s^−1^ for *syn*‐CI‐2 16, 5.2 × 10^−15^ cm^3^ molecules^−1^ s^−1^ for *anti*‐CI‐2 16, and 2.1 × 10^−15^ cm^3^ molecules^−1^ s^−1^ for CI‐3 21, the rate coefficient of the reaction of CH_2_OO with (H_2_O)_2_ (*k_4_*) of 5.4 × 10^−12^ cm^3^ molecules^−1^ s^−1^ for CI‐1 28, 2.6 × 10^−14^ cm^3^ molecules^−1^ s^−1^ for *syn*‐CI‐2 28, 1.6 × 10^−11^ cm^3^ molecules^−1^ s^−1^ for *anti*‐CI‐2 28, and 1.3 × 10^−13^ cm^3^ molecules^−1^ s^−1^ for CI‐3 15, the loss process of sCI was estimated. The monthly average [H_2_O], [(H_2_O)_2_], and temperature data (Supporting Information, Table S2) were obtained from UK Meteorological office chemistry transport model, STOCHEM (Dick Derwent, Personal communication). From the total sCI production and sCI loss, the steady‐state sCI concentrations were calculated for all three UK sites. Using the lower and upper limits of the rate coefficient from different literature for the unimolecular loss (0.2–115 s^−1^ for CI‐1, 20–250 s^−1^ for *syn*‐CI‐2, 60–250 s^−1^ for *anti*‐CI‐2, 250–369 s^−1^ for CI‐3), the loss due to the reaction with H_2_O (9.0 × 10^−17^ – 4 × 10^−15^ cm^3^ molecules^−1^ s^−1^ for CI‐1, 1.9 × 10^−19^ – 4 × 10^−15^ cm^3^ molecules^−1^ s^−1^ for *syn*‐CI‐2, 5.2 × 10^−15^ – 3.4 × 10^−14^ cm^3^ molecules^−1^ s^−1^ for *anti*‐CI‐2, 3.9 × 10^−17^ – 2.1 × 10^−15^ cm^3^ molecules^−1^ s^−1^ for CI‐3), and the loss due to the reaction with (H_2_O)_2_ (4.0 × 10^−12^ – 7.4 × 10^−12^ cm^3^ molecules^−1^ s^−1^ for CI‐1, 2.5 × 10^−12^ – 3.0 × 10^−12^ cm^3^ molecules^−1^ s^−1^ for *syn*‐CI‐2, 1.6 × 10^−11^ – 5.0 × 10^−11^ cm^3^ molecules^−1^ s^−1^ for *anti*‐CI‐2), a range of sCI concentrations were calculated.

### Site Selection

Data for the generation of the sCI levels were obtained from three different sites, dictated by the availability of data. The selected areas have a range of environments (e.g., kerbside, suburban, and rural). Sites are different, being impacted heavily by vehicles (e.g., Marylebone Road) through to being heavily impacted by VOCs from vegetation (e.g., Harwell). All sites are at ground level and have continuous hourly data of all the species required for the calculation. The site description is summarized in Table II.

**Table II:** Site Description

Site	Description	Type	Data Period
London Eltham	Situated in the London Borough of Greenwich and surrounded by a number of sports field and a golf course	Sub‐urban	2003–2012
London, Marylebone Road	Highly congested site in central London and is in close proximity to Regents Park	Kerbside	1998–2012
Harwell	Situated in the outskirts of Oxford and surrounded by agricultural fields with a wooded area 25 meters southeast of it.	Rural	2001–2012

## RESULTS AND DISCUSSION

Table III provides the estimations for sCI from the study at two urban and one rural sites. The highest concentration of sCI (330 ± 620 molecules cm^–^^3^) is found at the polluted urban site, Marylebone Road, whereas lower concentration is found at the rural site, Harwell (90 ± 100 molecules cm^–^^3^). The maximum sCI levels returned are 58,710 molecules cm^–^^3^ for Marylebone Road, 6350 molecules cm^–3^ for London Eltham, and 5080 molecules cm^–3^ for Harwell and correspond with the highest alkene levels (Marylebone Road: 158.6 ppbv; London Eltham: 5.9 ppbv; Harwell: 3.1 ppbv) and ozone levels (Marylebone Road: 9.2 ppbv; London Eltham: 24.5 ppbv; Harwell: 26.5 ppbv). Considering the range of unimolecular loss and the loss due to the reactions with H_2_O and (H_2_O)_2_, the concentrations of sCI are found in the range of 30–730 molecules cm^–^^3^ for Harwell, 100–2980 molecules cm^–3^ for Marylebone Road, and 40–940 molecules cm^–3^ for London Eltham. Thorough analysis of the measurement sites over the years with the most complete data sets were studied to gain a good representation of sCI production as possible. Only nine alkene measurement data were used in the estimation of sCI in this study, but, many other alkenes (e.g., 2‐methylpropene, styrene, 2‐methyl‐2‐butene) can exist in the urban and rural atmosphere that would lead to a greater sCI output. The availability of the alkenes data was sporadic, with occasions when data existed for only 1 or none are excluded from the calculation. So, the estimation values of sCI are considered to be lower estimates, and it is therefore vital to the understanding of the sCI‐oxidizing ability that in‐depth analysis of the more complex alkenes is pursued. A wider number of alkenes measured would lead to a better overall picture of the impact of sCI in the atmosphere, but the current analysis provides some important parameters that can inform future studies.

**Table III:** Average sCI Concentrations (in molecules cm^–3^) at Different Sites of UK for the Time Period of 1998–2012

	Harwell	Marylebone Road	London Eltham
sCI	90 ± 100	330 ± 620	110 ± 110
Maximum sCI	5080	58710	6350
Daytime sCI	100 ± 120	420 ± 810	130 ± 110
Nighttime sCI	80 ± 90	240 ± 310	90 ± 110
sCI^1^	(80 ± 100) to (730 ± 940)	(320 ± 610) to (2980 ± 5770)	(100 ± 110) to (940 ± 970)
sCI^2^	(30 ± 30) to (90 ± 110)	(100 ± 160) to (350 ± 640)	(40 ± 40) to (120 ± 120)
sCI^3^	(80 ± 100) to (140 ± 150)	(320 ± 600) to (490 ± 810)	(100 ± 100) to (180 ± 170)

Note: All concentrations values have been shown as average ± 1 SD for the whole data series. The concentrations of sCI^1^, sCI^2^, and sCI^3^ were calculated with considering lower and upper limits of *k*_2_, *k*_3,_ and *k*_4_ for different sCI (CI‐1, CI‐2, CI‐3), respectively.

**Figure 1:**
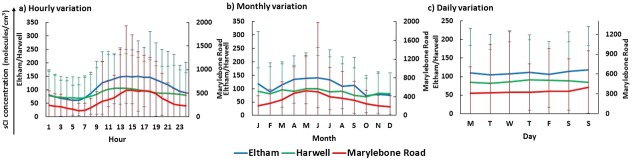
The hourly, monthly, and daily sCI concentrations calculated in the two urban sites and one rural site of UK. Note: The error bars represent ± 1 SD of the whole data series.

**Figure 2:**
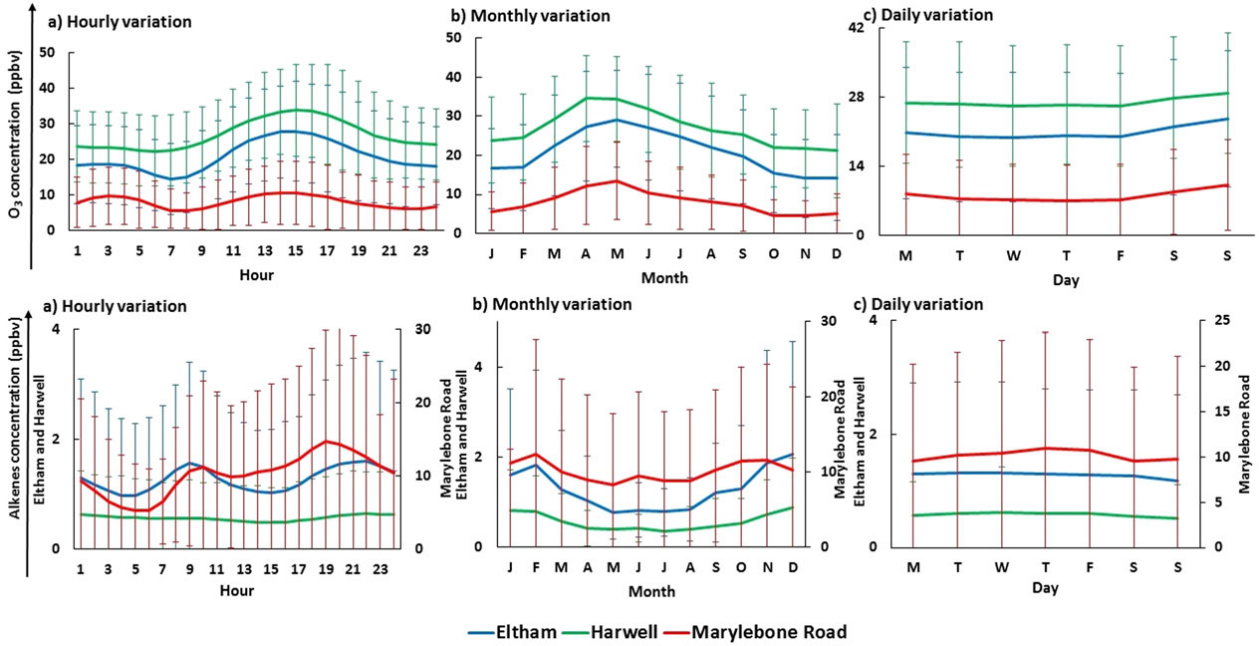
The hourly, monthly, and daily O_3_ and nine alkenes concentrations obtained from the NETCEN database for two urban sites and one rural site of UK. Note: The error bars represent ± 1 SD of the whole data series.

The average sCI concentrations over the time period of 1998–2012 for all three sites of UK (e.g., London Eltham, Marylebone Road, and Harwell) shows a consistent diurnal cycle with increasing levels during daylight hours, peaking just after midday before decreasing into the night (Fig. 1a). The main factor affecting the concentration of sCI is the production channel from VOCs + O_3_. The alkene levels show a diurnal cycle peaking during rush hours (9:00 am and 7:00 pm) for London Eltham and Marylebone Road sites when the emission of anthropogenic VOCs is at its greatest (see Fig. 2a). O_3_ follows a consistent diurnal pattern with maximums in the early afternoon (see Fig. 2a) due to the photochemical production, then decreases during the late afternoon before reaching its minimum level at night due to loss by the reaction with NO*_x_* 47 and by dry deposition onto surfaces 48; this trend is consistent with previous studies 49, 50, 51, 52. The higher concentrations of VOCs and O_3_ throughout the day promote sCI production, explaining the daytime highs calculated. The ratio of daytime to nighttime sCI is found to be 1.3, 1.8, and 1.4 for Harwell, Marylebone Road, and London Eltham sites, respectively.

A clear seasonal cycle of sCI concentrations peaking in May is observed for the three monitoring sites in the UK for the period of 1998–2012 (Fig. 1b). The variable levels of O_3_ is the main factor for the seasonal changes in sCI production. The changes in VOC throughout the year are not significant with slightly lower levels in the summer months (see Fig. 2b), but the higher levels of O_3_ in spring months (peak in April/May) (Fig. 2b) lead to an overall increase in sCI production in May. The lower levels of sCI are found throughout the winter months because of the lower O_3_ levels.

The sCI concentrations for all sites vary slightly throughout the week, as shown in Fig. 1c. It seems that the higher concentrations of sCI in the urban sites are found at the weekends compared with the weekdays. The ozone formation in urban areas are generally “VOC‐limited” and proportionately lower NO*_x_* emissions on weekends increase the VOC/NO*_x_* ratio, thus the nonlinear ozone photochemistry can generate more ozone on weekends than on weekdays 53, 54. The lower weekend concentrations of NO*_x_* also reduce the destruction of ozone by the reaction of O_3_ + NO → NO_2_ + O_2_, leading to higher weekend concentrations of ozone 55, which result in higher weekend sCI concentrations at UK urban sites. Contrary to UK urban sites, no significant differences between weekday and weekend sCI concentrations were found at the UK rural site Harwell because the site has no significant roads and thus more distant from the sources of NO*_x_* (e.g., vehicular traffic), thus the role of NO*_x_* for altering O_3_ concentrations between weekend and weekdays is minimal.

**Figure 3:**
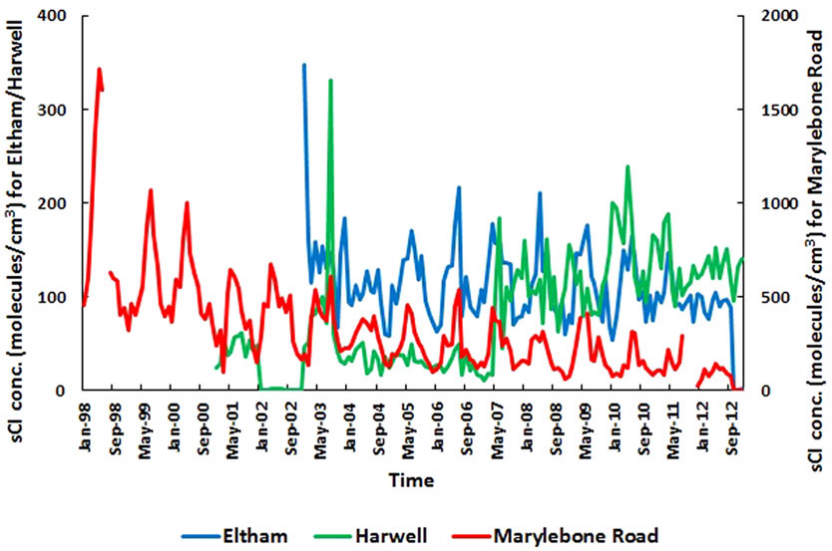
Average monthly variation of estimated sCI concentrations for three UK sites over the time series of 1998–2012.

The monthly average variation of sCI concentrations over the time series of 1998–2012 for the UK sites is shown in Fig. 3. One trend of significance in Fig. 3 is the overall decrease in sCI concentration from 1998 to 2012 for the Marylebone Road site. Over the 15 years, the high sCI concentration was found in 1998. A year‐to‐year decline occurs, and an overall decrease in 2012 observed because of the large decrease in the levels of anthropogenic VOCs. This pattern has arisen due to imposed legislation on VOC emissions especially from motor vehicles, paint, printing, and pharmaceutical products 56. The decreasing pattern can also be attributed to the stricter emission limits required to pass Motor of Transport (MOT) testing from 1998. Six percent of the 22 million light duty vehicles failed MOT inspection (98/99) due to excessive emissions 57, removing these vehicles reduced a large amount of VOC emissions that have an impact on the sCI concentrations during 1999–2000 (see Fig. 3). The legislation and government policies, e.g., 1999 Gothenberg protocol was signed by the UK, which was aimed to reduce VOC emissions by 40% from 1990 levels (by 2010). Given the urban location of the Marylebone Road and the high levels of traffic congestion experienced, it is not surprising that the implementation of the legislation has led to an overall decrease in sCI levels. No clear trend has been found for the London Eltham site, but for Harwell site, the sCI level is found to be higher during 2003 because of the significantly higher emissions of propene (0.83 ppbv). A steady increase of sCI level has been seen from 2007 due to the increasing pattern of the alkene levels (e.g., 0.37 ppbv in 2006, 0.49 ppbv in 2007, 0.51 ppbv in 2008) in Harwell site.

**Figure 4:**
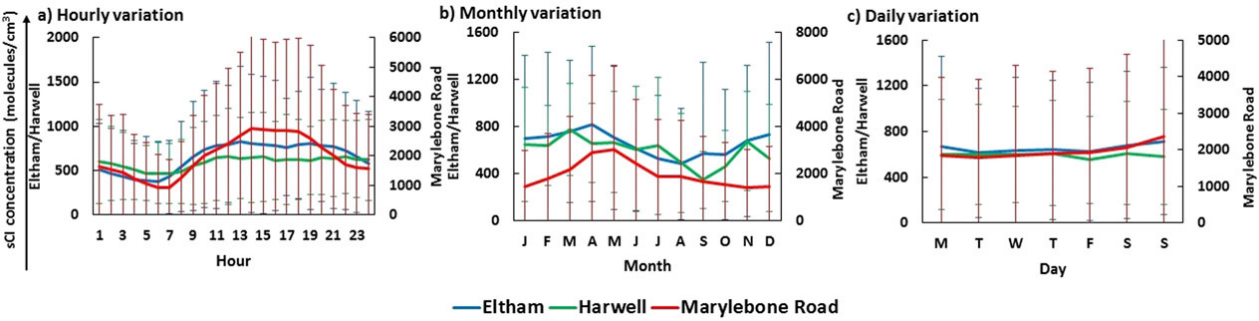
The hourly, monthly, and daily model sCI concentrations in the two urban sites and one rural site of UK. The error bars represents ± 1 SD of the whole data series.

A model calculation was performed with the study of the tailpipe exhausts from catalytic convertor equipped motor vehicles which was carried out in 1995 as part of the AQIRP detailing percentage composition of nonmethane hydrocarbons (NMHCs) from these emissions 58. The most consistent (most frequently reported) alkene data available in the NETCEN database is ethene, and it is these concentrations of ethene that have allowed extended alkene data to be modeled. From the AQIRP study, the total alkene composition is found to be 13.5% of the total NMHCs with ethene accounting for 3.68%. Fifty‐two alkenes were listed (see Supporting Information Table S3) in total with contributions ranging from 0.01% to 3.68%, and the nine alkenes used in the steady‐state approximation calculation of Criegee contributed ∼60% of the total alkenes. Given these percentages, a ratio of NMHC to ethene was used to calculate the concentration of all 52 alkenes based on a known concentration of ethene from the NETCEN database for the three UK sites. From these extended alkenes concentrations, total sCI levels for UK sites were calculated using Eq. (II). The rate coefficients of the formation of sCI from alkenes + O_3_ can be found in MCM website (http://mcm.leeds.ac.uk/MCM/search.htt). The sCI concentrations calculated using modeled alkene data have a similar diurnal cycle with peaks during the afternoon (see Fig. 4a), similar yearly cycle with peaks in May (see Fig. 4b) and similar weekly cycle with peaks in Sunday (see Fig. 4c) when compared with sCI concentrations calculated from nine measured alkenes data. However, the modeled values for total sCI production are five‐ to six‐fold higher (London Eltham: 660 ± 650 molecules cm^−3^, London Marylebone Road: 1960 ± 2390 molecules cm^−3^, and Harwell: 590 ± 440 molecules cm^−3^) than the estimated data. This is to be expected given the number of alkenes used in the model versus the available alkenes used in the estimation of sCI. Nonetheless, the magnitude of the difference could be of great significance, with increased levels of sCI in the atmosphere will increase their overall effect on the oxidizing cycle and have consequences for other atmospheric species. It is clear that this analysis is purely an example; it is impossible to say with certainty whether the ratios calculated using the AQIRP data are appropriate for the urban areas of UK or that the ratio method will be representative throughout the year, but the analysis can give a sensible way to estimate the unmeasured portion of the alkenes. However, adding in these approximate levels of known alkenes that are present in urban environments but not measured raises the estimated sCI significantly. Furthermore, emissions of naturally occurring alkenes, e.g., monoterpenes, sesquiterpenes, which are not accounted for, could have a significant additional impact on sCI levels, particularly in spring and summer months. It is clear, that if we want to fully understand the role of sCI in the troposphere, that more comprehensive measurements of alkenes are required.

**Figure 5:**
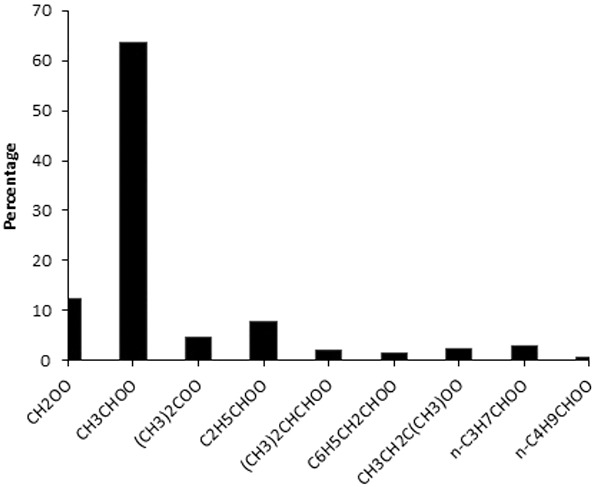
Percentage contributions of some dominant sCI to the total model sCI concentrations.

The contribution of individual modeled sCI species to the total modeled sCI concentrations has been investigated (see Fig. 5) and found that the two dominant sCI, CH_3_CHOO (dominating precursors: 2‐methyl‐2‐butene, trans‐2‐butene, cis‐2‐butene, trans‐2‐pentene, propene, cis‐2‐pentene, 4‐methyl‐trans‐2‐pentene, 3‐methyl‐trans‐2‐pentene) and CH_2_OO (dominating precursors: 2‐methylpropene, propene, styrene, ethene) contribute approximately 64% and 13%, respectively, to the total sCI concentration. In addition to these two species, C_2_H_5_CHOO (dominating precursors: *trans*‐2‐penetene, *cis*‐2‐pentene, *trans*‐3‐hexane, 2‐methyl‐2‐pentene) and (CH_3_)_2_COO (dominating precursors: 2‐methylpropene, 2‐methyl‐2‐pentene) account for approximately 8% and 4% of the total sCI concentrations. These results are different to previous percentage contribution results of sCI in eastern United States 59. This can be explained by the different emission scenarios of UK and U.S. sites, e.g., UK urban sites have smaller biogenic alkene sources, but larger biogenic emission sources are available in eastern United States and higher spring and summer temperatures which enhance their emission rates contributing ∼30% CH_2_OO (a significant contribution from isoprene) and ∼20% APINBOO (sCI from only α‐pinene) to the total sCI concentration. The reaction of (H_2_O)_2_ with CH_2_OO has the fastest rate of the reaction and has become the most important to the sCI loss rate 27, 31, which needs to be considered for reassessing the contribution of sCI. Thus, we excluded the contribution of CH_2_OO in the estimation of the total sCI and found that CH_3_CHOO, C_2_H_5_CHOO, and (CH_3_)_2_COO can contribute 73, 9, and 5%, respectively to the total sCI concentration.

The impact of sCI in the atmosphere has been investigated with considering two significant reactions of sCI with NO_2_ and SO_2_. NETCEN database gives the annual average NO_2_, SO_2_, O_3_ levels of 50.0, 3.1, and 7.6 ppbv, respectively, for Marylebone Road in 2012. If the global urban areas is of 3.5 million km^2^ 60, the average concentration of OH in urban areas is of 1 × 10^6^ molecules cm^−3^, the average day–night boundary layer height is of 550 m (typical urban boundary layer is 1 km during daytime and 100 m during nighttime) considering the surface level sCI, NO_2_, SO_2_, O_3_ concentrations of Marylebone Road, which are constant throughout the urban boundary layer, and the rate coefficients of the reactions of sCI + NO_2_ and sCI + SO_2_ are of *k*_sCI+NO2_ ∼2.0 × 10^−12^ cm^3^ molecules^−1^ s^−1^ and *k*_sCI+SO2_ ∼4.0 × 10^−11^ cm^3^ molecules^−1^ s^−1^, respectively, a back‐of‐the‐envelope calculation gives the upper limit oxidation rates of NO_2_ + sCI (22.4 Gg/yr) and SO_2_ + sCI (37.6 Gg/yr) to produce NO_3_ and SO_3_ in urban areas, which are notable comparing with the most conventional oxidation rates of NO_2_+O_3_ (50.8 Tg/yr) and SO_2_ + OH (0.64 Tg/yr) in urban areas. The further reactions of NO_3_ and SO_3_ can produce alkyl nitrates and H_2_SO_4_, respectively, which can form organic aerosol in the urban areas. The high levels of aerosols in the urban areas can negatively affect human health and local air quality. They can have a net cooling effect on climate by changing the earth's energy budget by scattering and absorbing the radiation or acting as cloud condensation nuclei. The consequences of sCI concentrations in urban areas are important, but it is an area of much needed study and more research is required to gain a more comprehensive understanding of the potential impacts of sCI.

## CONCLUSIONS

The steady‐state method has been applied to estimate the concentrations of sCI at two urban sites (London Eltham, Marylebone Road) and one rural site (Harwell) in the UK over the period 1998–2012 from trace gas data provided by the NETCEN database. All sites have higher sCI levels in spring months and lower in winter months, with peaks around May. Higher levels of O_3_ in May result in an overall increase in the sCI production. A clear diurnal cycle of sCI peaking just after midday was found for all sites. Comparatively lower weekday sCI concentrations are seen than weekend in UK urban sites. The concentration of anthropogenic VOCs saw a dramatic decrease over the years due to EU legislation and was accompanied by an overall decrease in sCI production over the years for urban sites. Increased alkenes data set in model calculation resulted in a significant increase in sCI, so our estimated sCI concentrations are low estimates; and overall sCI concentrations in these areas have the potential to be higher than initially thought. The two sCI, CH_3_CHOO (64%) and CH_2_OO (13%), are the dominant in the UK urban atmosphere among all contributing sCI.

## Supporting information

Disclaimer: Supplementary materials have been peer‐reviewed but not copyedited.

Supplementary Material
