# Supplementary material for: An Estimation of the Levels of Stabilized Criegee Intermediates in the UK Urban and Rural Atmosphere Using the Steady‐State Approximation and the Potential Effects of These Intermediates on Tropospheric Oxidation Cycles
Source: Int J Chem Kinet. 2017 Jun 12;49(8):611–21. doi: 10.1002/kin.21101 (PMC5519938; doi:10.1002/kin.21101)
Supplement: Supplementary file 1 — Supplementary Material [file KIN-49-611-s001.docx]

### An estimation of the levels of stabilized Criegee intermediates in the UK urban and rural atmosphere using the steady state approximation and the potential effects of these intermediates on tropospheric oxidation cycles

M. Anwar H. Khan,^1^ William C. Morris,^1^ Matthew Galloway,^1^ Beth M. A. Shallcross,^2^ Carl J. Percival,^3^ Dudley E. Shallcross^1^

**Table S1** Selected alkenes and the rate coefficients for the ozonolysis reactions used for calculating sCI production

| Alkenes | Molecular formula | Rate coefficient^a^ (cm^3^molecules^-1^s^-1^) |
| --- | --- | --- |
| 1,3-Butadiene | C_4_H_6_ | 1.34 × 10^-14^exp(-2283/T) |
| 1-Butene | C_4_H_8_ | 3.36 × 10^-15^exp(-1744/T) |
| 1-Pentene | C_5_H_10_ | 9.97 × 10^-18^ |
| Ethene | C_2_H_4_ | 9.14 × 10^-18^exp(-2500/T) |
| Isoprene | C_5_H_8_ | 7.86 × 10^-15^exp(-1913/T) |
| Propene | C_3_H_6_ | 5.51 × 10^-15^exp(-1878/T) |
| *cis*-2-Butene | C_4_H_8_ | 3.22 × 10^-15^exp(-968/T) |
| *trans*-2-Butene | C_4_H_8_ | 6.64 × 10^-15^exp(-1059/T) |
| *trans*-2-Pentene | C_5_H_10_ | 1.59 × 10^-16^ |

Notes: ^a^Details can be obtained using species name and search facility on MCM website (<http://mcm.leeds.ac.uk/MCM/search.htt>).

**Table S2** Monthly average temperature, water, and water dimer for 1998 (extracted from UK Meteorological office chemistry transport model, STOCHEM) used for calculating sCI concentration

| Month | Temperature (^o^K) | Water density (molecules/cm^3^) | Water dimer density  (molecules/cm^3^) |
| --- | --- | --- | --- |
| January | 276.0 | 1.71 × 10^17^ | 1.21 × 10^14^ |
| February | 278.3 | 2.09 × 10^17^ | 1.67 × 10^14^ |
| March | 275.8 | 1.68 × 10^17^ | 1.18 × 10^14^ |
| April | 275.4 | 1.69 × 10^17^ | 1.20 × 10^14^ |
| May | 280.1 | 2.09 × 10^17^ | 1.58 × 10^14^ |
| June | 281.0 | 2.36 × 10^17^ | 1.95 × 10^14^ |
| July | 282.2 | 2.65 × 10^17^ | 2.36 × 10^14^ |
| August | 283.1 | 2.73 × 10^17^ | 2.44 × 10^14^ |
| September | 283.3 | 2.81 × 10^17^ | 2.56 × 10^14^ |
| October | 279.0 | 2.04 × 10^17^ | 1.55 × 10^14^ |
| November | 276.7 | 1.76 × 10^17^ | 1.25 × 10^14^ |
| December | 276.6 | 1.77 × 10^17^ | 1.26 × 10^14^ |

**Table S3** Total 52 alkenes used in sCI modelled data and their percent contributions

| Compound | % emissions relative to total NMHCs | % emissions relative to total alkenes |
| --- | --- | --- |
| ethene | 3.68 | 27.34 |
| 2-methylpropene | 2.95 | 21.92 |
| propene | 2.64 | 19.61 |
| styrene | 0.42 | 3.12 |
| 1-butene | 0.38 | 2.82 |
| 1,3-butadiene | 0.36 | 2.67 |
| 2-methyl-2-butene | 0.35 | 2.60 |
| *trans*-2-butene | 0.27 | 2.01 |
| 2-methyl-1-butene | 0.24 | 1.78 |
| *cis*-2-butene | 0.24 | 1.78 |
| isoprene | 0.19 | 1.41 |
| *trans*-2-pentene | 0.17 | 1.26 |
| 1-nonene | 0.16 | 1.19 |
| propadiene | 0.15 | 1.11 |
| 1-pentene | 0.1 | 0.74 |
| *cis*-2-pentene | 0.09 | 0.67 |
| cyclopentadiene | 0.08 | 0.59 |
| 3-methyl-1-butene | 0.08 | 0.59 |
| 4-methyl-*trans*-2-pentene | 0.08 | 0.59 |
| 3-methyl-*trans*-2-pentene | 0.07 | 0.52 |
| 3-methyl-*cis*-2-pentene | 0.06 | 0.45 |
| *trans*-2-hexene | 0.05 | 0.37 |
| 2-methyl-2-pentene | 0.05 | 0.37 |
| 1-hexene | 0.05 | 0.37 |
| cyclopentene | 0.05 | 0.37 |
| 4-methyl-1-pentene | 0.05 | 0.37 |
| 2-methyl-1-pentene | 0.04 | 0.30 |
| 2-methyl-2-hexene | 0.04 | 0.30 |
| 3-methyl-1-pentene | 0.03 | 0.22 |
| *trans*-3-hexene | 0.03 | 0.22 |
| 4-methyl-*cis*-2-pentene | 0.03 | 0.22 |
| *cis*-2-hexene | 0.02 | 0.15 |
| 1-octene | 0.02 | 0.15 |
| *trans*-3-heptene | 0.02 | 0.15 |
| *cis*-2-heptene | 0.02 | 0.15 |
| *trans*-2-heptene | 0.02 | 0.15 |
| 3,4-dimethyl-1-pentene | 0.02 | 0.15 |
| 2,4,4-trimethyl-1-pentene | 0.02 | 0.15 |
| *trans*-4-octene | 0.01 | 0.07 |
| 2,4,4-trimethyl-2-pentene | 0.01 | 0.07 |
| 1-heptene | 0.01 | 0.07 |
| cyclohexene | 0.01 | 0.07 |
| 3,3-dimethyl-1-butene | 0.01 | 0.07 |
| *cis*-3-hexene | 0.01 | 0.07 |
| 3-methyl-1-hexene | 0.01 | 0.07 |
| *trans*-2-octene | 0.01 | 0.07 |
| *cis*-2-octene | 0.01 | 0.07 |
| 3-methyl-cyclopentene | 0.01 | 0.07 |
| 3-methyl-*trans*-3-hexene | 0.01 | 0.07 |
| 3-ethyl-*cis*-2-pentene | 0.01 | 0.07 |
| 2,3-dimethyl-2-pentene | 0.01 | 0.07 |
| 1-methyl-cyclopentene | 0.01 | 0.07 |
